# Our (Mother’s) Mitochondria and Our Mind

**DOI:** 10.1177/1745691617718356

**Published:** 2017-09-22

**Authors:** Peter Kramer, Paola Bressan

**Affiliations:** Department of General Psychology, University of Padua, Italy

**Keywords:** mitochondria, free radicals, aging, Alzheimer’s disease, Parkinson’s disease, depression, ketogenic diet

## Abstract

Most of the energy we get to spend is furnished by mitochondria, minuscule living structures sitting inside our cells or dispatched back and forth within them to where they are needed. Mitochondria produce energy by burning down what remains of our meal after we have digested it, but at the cost of constantly corroding themselves and us. Here we review how our mitochondria evolved from invading bacteria and have retained a small amount of independence from us; how we inherit them only from our mother; and how they are heavily implicated in learning, memory, cognition, and virtually every mental or neurological affliction. We discuss why counteracting mitochondrial corrosion with antioxidant supplements is often unwise, and why our mitochondria, and therefore we ourselves, benefit instead from exercise, meditation, sleep, sunshine, and particular eating habits. Finally, we describe how malfunctioning mitochondria force rats to become socially subordinate to others, how such disparity can be evened off by a vitamin, and why these findings are relevant to us.

Some people expend tremendous energy merely to be normal.

—Albert Camus, *Notebooks: 1942–1951*

Some of us go about life with less energy than others. For many suffering from one of the myriad neurodegenerative, neurological, cognitive, and mental disorders humans are blessed with, such lack of energy is much more than a metaphor. Most of the energy we get to spend is delivered by minuscule living structures dispatched back and forth or docked in crucial districts inside our cells, *mitochondria*, by burning down what remains of our meal after we have digested it. From this material, mitochondria produce energy for the cells to run on—in a grander view, energy for the body and brain to run on. Thus, how strong and active and ultimately happy we can be depends a great deal on how well this lowly machinery is working.

We have previously presented a broad overview, in plain English, of how invading microbes, viruses, and other selfish entities have to various extents become integrated into people’s body and brain and actively shape human behavior (P. Kramer & Bressan, 2015). Here we extend our overview to the descendants of one spectacularly successful bacterial invasion: mitochondria. We review how mitochondria have retained various bacterial characteristics and a small amount of independence from humans, almost like enslaved but separate creatures; why they can have powerful effects, both good and bad, on people’s mental health; and what can be done to increase the good and decrease the bad.

## Love in a Cold Climate

Life on earth has existed for around 4 billion years (Dodd et al., 2017). For the first 2 billion, all creatures alive were single cells with no nucleus (Dacks et al., 2016). Then—according to a leading theory (Martin & Müller, 1998; Martin, Neukirchen, Zimorski, Gould, & Sousa, 2016)—certain bacteria invaded certain archaea, members of a separate domain of microbes. At the time, these archaea lived around volcanic ridges on the ocean floor and fed on carbon dioxide and on hydrogen from submarine hydrothermal vents. The bacteria lived in the vicinity, fed on organic compounds, and discharged as waste products precisely the carbon dioxide and hydrogen that the archaea made use of. Microbes do sometimes break into one another and the result is rarely a happy marriage. After tolerating the bacteria for a while, however, the invaded archaea evolved to take advantage of them and ended up integrating them for good. In the process, the bacteria became mitochondria, the milk cows of their hosts, and shed most of their genetic material (Lane, 2015; Lane & Martin, 2010). Thanks to their new milk cows, the hosts had plenty of energy ready for use and could afford to pick up parts of the discarded material, build a kind of control center out of it—a cell “nucleus”—and evolve from prokaryotes (organisms made of one cell with no nucleus) into eukaryotes (organisms made of one or more cells with a nucleus, like us).

Regardless of whether eukaryotes emerged this way (Lane, 2015; Martin & Müller, 1998) or a different one (Koonin, 2015), *all* eukaryotes contain either bacteria-like mitochondria with bacterial DNA or, in exceptional cases, remnants of it (Martin et al., 2016). Prokaryotes are sexless and reproduce by making copies of themselves. Occasionally, they exchange chunks of genetic material with one another (Hanage, 2016) and hand down the resulting mix to the next generation via cloning. Eukaryotes, on the other hand—including virtually all of those that can clone themselves—reproduce sexually at least some of the time (Speijer, 2016). Sex mixes up genes extraordinarily well and this bears various potential advantages. It forces parasites to constantly readapt (Tooby, 1982), for example, and it does in fact diminish their ability to break through host defenses (Auld, Tinkler, & Tinsley, 2017). Sexual reproduction involves passing on not only genes but also all the rest of the fertilized egg, including live mitochondria. Yet, although eukaryotic parents mix their genes, they avoid mixing their mitochondria; in the overwhelming majority of cases only one parent—almost always the mother—passes them on (Breton & Stewart, 2015). Why this should be so continues to be debated (see next section), but given that it is the case and that only eukaryotes have mitochondria, only eukaryotes have sex, and nearly all eukaryotes have sex at least some of the time, many agree that the evolution of sex is likely to have a lot to do with mitochondria (Lane, 2015; Speijer, 2016).

Sexual reproduction complicates the lives of those involved considerably. Unlike prokaryotes, eukaryotes usually do not exchange genetic material with just any other eukaryote; they need to find a suitable mate that must be of the same species, opposite sex, and either won over to collaborate or forced to do so. Meanwhile they may face the risks of retribution (for example, in case of attempted rape), of contracting a sexually transmitted disease, and—especially if they are human females—of potentially fatal complications during labor. To ensure that genes and mitochondria are nevertheless passed on from one generation to the next, individuals must therefore somehow be compensated—“bribed,” in our species, with “intense erotic pleasure; presumably mild pleasure is not enough” (Lane, 2005, p. 206).

Most of our DNA (including some 20,000 genes) sits in the nucleus of each of our cells, and this nuclear DNA regulates much of how our mitochondria function (Mattson, Gleichmann, & Cheng, 2008). Outside the nucleus, however, mitochondria have retained some DNA of their own (including, in humans, 37 genes) and this gives them a small degree of independence. Human mitochondrial DNA, unlike nuclear DNA, is passed on only from mothers (Pyle et al., 2015). So, any mutations in mitochondrial DNA are weeded out of the gene pool only if they harm women and not if they harm men; for mitochondria, men are just about dead ends, and their future makes no difference to mitochondrial future. Men and women have much in common, and what is harmful to men is usually also harmful to women, but there is at least one exception.

From digested food, mitochondria produce both adenosine triphosphate (ATP)—a chemical that cells use as fuel—and heat, which can be spent to maintain body temperature. The more heat, the less ATP and vice versa. People in cold climates are more likely than those in hot ones to have inherited mitochondria that produce relatively much heat (Ruiz-Pesini et al., 2000). For men, however, there is a catch, because they host one cell type that happens to be particularly sensitive to a loss in ATP: sperm. To be fast, sperm cells need to be small. Typically they accommodate only up to a few dozen mitochondria (there to provide swimming energy), whereas other body cells carry on average 300 to 400 and egg cells, which are huge, about 100,000 (Lane, 2015). If those few sperm mitochondria do not make enough ATP, fertility diminishes. But because only women pass on their mitochondria and women do not carry sperm, the genes of inefficient sperm mitochondria may become more frequent than they otherwise would have. Indeed, mitochondrial DNA associated with reduced male fertility has been found to be more common in men who live in colder climates than in those who live in warmer ones (Ruiz-Pesini et al., 2000): in Swedes (22%) more than in Germans (12%) and in Germans more than in the Druz from the Middle East (4%).

Unlike a cell’s nucleus that has just one set of DNA, each mitochondrion can carry several (e.g., Cavelier, Johannisson, & Gyllensten, 2000)—meaning up to hundreds or thousands of copies per cell. Each set is used in the production of the same proteins. Should one set no longer function properly, due for example to accumulation of mutations, another can compensate and this renders mitochondria much more resilient than they otherwise would be. The number of copies of mitochondrial DNA is not the same for everyone, though, and this is not an easy number to interpret. A smaller one should stand for reduced mitochondrial efficiency, and on such grounds reduced efficiency of body and brain (Cao, Zhao, Zhou, Chen, & Yang, 2012). Among healthy elderly women, in fact, those who do worse in a cognitive abilities test have fewer copies than those who do better (J. W. Lee, Park, Im, Kim, & Lee, 2010), and those who are depressed have fewer copies than those who are not (Kim, Lee, Kang, Kim, & Lee, 2011). However, healthy young adults with a history of either depression, anxiety, substance use disorder, childhood parental loss, or maltreatment have more DNA copies than people without such a history (Tyrka et al., 2016). Arguably, this increase could be an early compensatory response to adversity that is progressively overwhelmed as one gets older or sicker (Tyrka et al., 2016).

## Air, Fire, and Dangerous Things

### Mitochondria corrode

To produce ATP, mitochondria burn down digested food with the help of the oxygen we inhale. In the process, they dump several waste products: carbon dioxide (which eventually we can exhale), water (which eventually we can pee out), and *free radicals*. Free radicals are chemicals that are highly corrosive, first and foremost to the mitochondria themselves but also to their hosts. Indeed, how healthy a cell is can be judged by the shape of its mitochondria: They are tubular when all is well, turn into donut forms under the stress of too many free radicals, and into blobs when damage is irreversible. In a vicious cycle, the more its shape departs from normal, the more free radicals the mitochondrion produces (Ahmad et al., 2013).

The fact that mitochondria generate free radicals has led to an interesting idea about why much of the genetic material of the bacterial ancestors of mitochondria ended up in their host cell’s nucleus: Not only is that material useful to the cell, but in the nucleus it is also better protected from free radicals than in the mitochondria themselves (Speijer, 2016). The genes left in the mitochondria keep accumulating mutations and are quite tarnished by the time people reach reproductive age; yet these genes do not appear to be passed to the next generation in their damaged state. To solve the conundrum, it has been proposed that egg cells carry solely fresh, unspoiled “template mitochondria” that they do not put to use but just keep in storage (Allen, 1996; Allen & de Paula, 2013; supporting evidence in de Paula, Lucas, Agip, Vizcay-Barrena, & Allen, 2013). Egg cells can afford this extravagance because they mostly just stay put, and the little energy they need they can get from much less efficient sources than mitochondria or from the mitochondria of nearby cells in the ovary.

Sperm cells, whose only life plan is to win a swimming race, are forced instead to get as much mileage as possible out of their own mitochondria, at the cost of getting them marred by free radicals in the process. It is interesting that sperm mitochondria turn out to be already tagged with a kiss-of-death protein from the outset and are demolished straight off upon encountering the egg’s destruction machinery (Sutovsky et al., 1999). Thus both parents seem to be in (unusual) genetic agreement that only maternal mitochondria will serve as a template for all of the future baby’s. As Allen’s (1996) influential theory goes, most newly molded mitochondria are immediately activated and pressed into service, but in female embryos some are stored away in the developing ovaries in their pristine state, ready for the next generation—immortal, in a way— quite a good reason why exclusively maternal inheritance of mitochondria may have arisen. Tellingly, mice that have been forced to inherit mitochondria from both parents turn out to have physical, emotional, and cognitive problems (Sharpley et al., 2012).

To prevent as much as possible the damage (technically, oxidation) brought forth by free radicals, our mitochondria either manufacture antioxidants or use those we consume with our meals (Fraunberger, Scola, Laliberté, Duong, & Andreazza, 2016). Yet not all free radicals can be neutralized, and making the best of a bad predicament the body manages to put the remaining ones to good use. For example, activation of our immune system typically causes an increase in free radicals and vice versa (López-Armada, Riveiro-Naveira, Vaamonde-García, & Valcárcel-Ares, 2013), and both mechanisms help kill parasites. Free radicals that likely stem from sources other than mitochondria serve also as signaling molecules in learning and memory (Kishida & Klann, 2007). Thus, whereas carrying a high level of free radicals is bad, so is trying to eliminate them completely (Ristow & Schmeisser, 2011). In fact, although antioxidant supplementation can be beneficial in some groups of people, in others it appears harmful and can even hasten death (Villanueva & Kross, 2012).

When injury cannot be avoided, damaged mitochondria can be repaired (Boesch et al., 2011). If the repair mechanisms fail, parts still in working order can often be recycled. To that purpose, pairs of malfunctioning mitochondria fuse together and come apart again; in the process, the best components of each pair are left in one mitochondrion and the other is targeted for destruction (Youle & van der Bliek, 2012). Yet this is not always enough, and cells that harbor too many dysfunctional mitochondria, or have incurred too much free-radical damage themselves, become dysfunctional too. To avoid reproducing—and make way for healthier substitutes—such cells instruct their own mitochondria to kill them and to commit suicide in turn. Unfortunately, on the large scale of things the benefits of this intervention are only temporary.

A place where mitochondrial trouble occurs frequently is the brain. Although its mass is a mere 2% of the body’s total, the human adult brain uses up to 25% of the body energy (Herculano-Houzel, 2012). By comparison, it is only 13% in other primates and 2% to 8% in the vast majority of vertebrates (Mink, Blumenschine, & Adams, 1981). A resting cortical neuron turns out to consume 4.7 billion ATP molecules per second; a resting human brain goes through nearly 6 kilograms of them per day (Zhu et al., 2012). Inevitably, brain mitochondria produce a lot of free radicals and this contributes to making the brain itself particularly vulnerable to free-radical damage—or “brain rust” as it has been called (de Oliveira, Ferreira Lima, & El-Bachá, 2012). Cognitive symptoms in Alzheimer’s disease are preceded by energy deficits in neurons (Kapogiannis & Mattson, 2011), and mitochondrial dysfunction is associated with Parkinson’s disease (Haddad & Nakamura, 2015) and is enough to cause Parkinson-like symptoms in rats (Mattson et al., 2008).

As people age, their blood flow diminishes and the brain gets progressively less blood and thus less glucose and oxygen for mitochondria to make ATP with. Eventually (as argued in de la Torre, 2002), brain mitochondria no longer meet the normal energy demand and neurons degenerate, leading to dementia—whose prevalence, between the ages of 55 and 90, increases 400-fold (Harvey, Skelton-Robinson, & Rossor, 2003; Prince et al., 2015). The most disrupted region in Alzheimer’s disease is the hippocampus—an area, involved in memory, that compared to most other areas of the brain contains remarkably low levels of a protein that helps oxygen diffuse to the mitochondria (Burmester, Weich, Reinhardt, & Hankeln, 2000; Moens & Dewilde, 2000). The most disrupted region in Parkinson’s disease is the substantia nigra pars compacta—an area involved in motor planning, each of whose large and highly interconnected neurons is estimated to house an impressive 2 million mitochondria, with a mandatory replacement rate of over 20 new mitochondria every minute (Shlevkov & Schwarz, 2017). Only the neurons in the pars compacta are affected in Parkinson’s disease; the much smaller, much less branched, much less energy-demanding ones in nearby regions are spared (Pacelli et al., 2015). All of these are unlikely to be coincidences.

### Mitochondria multitask

Whatever the price, the nervous system cannot possibly function without mitochondria (Markham, Bains, Franklin, & Spedding, 2014; Mattson et al., 2008). Neurons relay information by firing and mitochondria provide the energy for this. Transported by miniature motors along tubular tracks, they travel extensively inside the neuron to furnish ATP where it is needed (Sheng & Cai, 2012). Although neurons use up ATP even when all they are doing is basic housekeeping, energy is in highest demand at synapses, where most of the action occurs. Mitochondria are transferred and docked there only when they are in good shape. As soon as they become unfit and in need of replacement they are promptly destroyed, either on the spot (Ashrafi, Schlehe, LaVoie, & Schwarz, 2014) or after having been removed and transported away (Lin & Sheng, 2015). Poor quality control at this stage is linked to neurodegenerative diseases (Martinez-Vicente, 2017); poor transport of mitochondria precedes the onset of Alzheimer’s and Parkinson’s diseases (Correia, Perry, & Moreira, 2016; Shlevkov & Schwarz, 2017) and might play a part in schizophrenia and depression as well (Deheshi, Pasqualotto, & Rintoul, 2013).

Every time it fires, a neuron is invaded by sodium ions. If enough of them have gotten inside, channels are unblocked that let calcium ions in as well. Both types of ions must be removed before the neuron can fire again; mitochondria give a hand by temporarily locking calcium away and dispensing energy for other structures in the neuron to do so. When somewhere in the neuron ATP runs low or calcium gets in, approaching mitochondria are disengaged from the transport system so that they can do their job at that particular site (Sheng & Cai, 2012). However, an inordinate accumulation of calcium in mitochondria causes them to kill both their host neuron and themselves—which can happen when firing is excessive, as in epileptic seizures (Delorenzo, Sun, & Deshpande, 2005). As shown in vitro, the cold sore virus (herpes-simplex virus type 1) can boost neuronal calcium levels while at the same time impeding mitochondria from killing their host. The raised calcium levels prevent the transport system from taking mitochondria as passengers and possibly allow the virus to take a ride instead, speeding up its spread through the brain (T. Kramer & Enquist, 2012). By age 60, up to 90% of the population in most countries is infected with the cold sore virus; in individuals with both a genetic predisposition and a weak immune system, its presence has been linked to neurodegeneration (Caggiu et al., 2016; Itzhaki, 2016) and schizophrenia (Carter, 2009).

When all is well, a rise in calcium levels can set off a chain of reactions that renders the neuron more responsive to future stimulation; a reduction does the opposite. Calcium affects this so-called synaptic plasticity in all kinds of manners, not least by sparking the release of brain-derived neurotrophic factor (BDNF for short), a protein that stimulates the growth and repair of active neurons and synapses (Markham et al., 2014). Mitochondria affect synaptic plasticity both ways: On the one hand, prompted by BDNF, they supply the energy needed to strengthen neuronal connections; on the other, by delivering the same enzymes they use when forced to kill off their host cell, they can prune connections away (Jeanneteau & Arango-Lievano, 2016; Markham et al., 2014).

At this point, one must expect mitochondria to be entangled in about every possible mental process—especially those that require a great deal of firing. Indeed, a particular area of monkeys’ prefrontal cortex that is involved in working memory—a capacity that is heavily used all the time—harbors resident mitochondria near most of its synapses. The larger the number of properly shaped (and the smaller the number of donut-shaped) mitochondria one finds there, the better monkeys do on a working memory test (Hara et al., 2014). As an interesting aside, the formation of donuts can be prevented by antioxidants, and estrogen is a formidable one. If female monkeys’ ovaries are removed, a maneuver that cuts off estrogen and induces menopause, the number of misshaped mitochondria increases nearly by half and monkeys become much worse on the test. Both changes are reversed by cyclic injections of estrogen—suggesting that it is, at least partly, by restoring mitochondrial health that hormone replacement therapy may improve cognitive functioning (Hara et al., 2014; Sherwin, 1988). Curiously, compared to premenopausal ones, monkeys that have transitioned naturally into menopause harbor actually more, rather than fewer, good mitochondria near their synapses. This may reflect a compensatory mechanism against the mitochondrial dysfunction expected with natural female aging (Hara et al., 2014).

Mitochondria do not merely help regulate calcium levels and provide energy. Their ATP can also be used as a signaling molecule in its own right, for example as a neurotransmitter. Adenosine triphosphate (ATP) can be stripped of one or all three of its phosphate groups and become, respectively, adenosine diphosphate (ADP) or simply adenosine, and both these substances work as signaling molecules too. Dysregulation of the receptors of either ATP, ADP, or adenosine has been implicated in disorders ranging from a reduced urge to empty one’s bladder (whose stretching releases ATP: Cook & McCleskey, 2000) to depression and schizophrenia (Krügel, 2016) and the early stages of Alzheimer’s and Parkinson’s disease (Burnstock, 2016). As a neurotransmitter, ATP can leave cells only in a controlled way, usually in small quantities. As a result of injuries such as a cut, however, cells can rupture and spill a large amount of it. In such occasions ATP acts as a sentinel by activating nearby receptors on the outside of cells that function like alarm bells; we feel this as pain and know we have been hurt and need to take action (Wirkner, Sperlagh, & Illes, 2007).

ATP and its derivatives are not the only signaling molecules that mitochondria produce. Mostly in the ovaries, testes, and adrenal gland, they derive the precursor of steroid hormones like estrogen and testosterone by partially burning down cholesterol (Rossier, 2006). In the same way, mitochondria even manufacture small quantities of steroids, called neurosteroids, inside neurons. Neurosteroids have fast local effects on the brain whereas other steroids have slower systemic ones (King, 2013). Steroids and neurosteroids affect brain and behavior in a wide range of ways. Some neurosteroids can affect how much calcium enters the neuron, and this, in turn, has an impact on learning and memory, the risk of epileptic seizures, and mental health more generally (King, 2013; Korinek et al., 2011).

After use, or if present in excess, hormones and neurotransmitters tend to be stored away or broken down. The breaking down is again partly accomplished by mitochondria, by way of two enzymes of their own: MAO-A and MAO-B. Both metabolize, and thus curtail, critical neurotransmitters such as serotonin, dopamine, and noradrenalin (Buckholtz & Meyer-Lindenberg, 2008). Low levels of these enzymes during development—because of faulty genes on the X-chromosome—have been associated with abnormal behavior, and in the case of the *MAOA* gene most notably with aggressiveness, in both mice and men. Female mammals have two X-chromosomes, and if one is defective the other can (at least partially) compensate for it. Males, however, have only one X-chromosome and if this is defective they are stuck with it. If one directly knocks out the *MAOA* gene during development, as has been done in mice, MAO-A is no longer produced, serotonin and noradrenalin levels in the brain rise, and aggressiveness goes up (Buckholtz & Meyer-Lindenberg, 2008). In Western society, about one third of men carry a less active variant of the *MAOA* gene. Compared to those with the more active variant, these people are more inclined to retaliate when wronged, even when such retaliation comes at a cost to them (McDermott, Tingley, Cowden, Frazzetto, & Johnson, 2009). Eight men from the same Dutch family, studied because their *MAOA* gene did not work at all, showed violent outbursts following hardly any provocation at all (Brunner, Nelen, Breakefield, Ropers, & van Oost, 1993; Brunner, Nelen, van Zandvoort, et al., 1993). Some of them had engaged in an assortment of criminal behaviors, including arson, assault, attempted murder, and attempted or successful rape of family members.

### Mitochondria derange

Given the multiple first-rate jobs that mitochondria do in the nervous system, it is hardly accidental that their malfunctioning has been associated with virtually every mental or neurological affliction on earth, including chronic psychological stress and fatigue, cognitive deficits, Alzheimer’s and Parkinson’s disease, anxiety, depression, bipolar disorder, schizophrenia, autism, multiple sclerosis, and Down syndrome (Arbuzova, Hutchin, & Cuckle, 2002; Burroughs & French, 2007; Jeanneteau & Arango-Lievano, 2016; Kaplan, Rucklidge, Romijn, & McLeod, 2015; Manji et al., 2012; Morris & Berk, 2015; Toker & Agam, 2015; Valenti, de Bari, De Filippis, Henrion-Caude, & Vacca, 2014). People diagnosed with an inherited disorder that affects all of their mitochondria have been reported to carry a 50% to 70% probability—more than two or three times as high as ordinary people’s—of developing a psychiatric condition at some point in life (Fattal, Link, Quinn, Cohen, & Franco, 2007; Inczedy-Farkas et al., 2012; Mancuso et al., 2013; for a review, see Kaplan et al., 2015). Not unexpectedly, given its effects on mitochondria, dysregulation of either calcium or BDNF has been implicated in numerous mental afflictions too (Autry & Monteggia, 2012; Markham et al., 2014). Mitochondrial malfunctioning also arouses the immune system (Markham et al., 2014), and an activated immune system drives mitochondria to produce even more free radicals (López-Armada et al., 2013). A chronically activated immune system is so common among people suffering from mental illness that this has become the focus of an entire field of research (Pariante, 2016).

Considering all of the above, it should come as no surprise that mental disorders are apt to hinge together (Devaraju & Zakharenko, 2017). For example, schizophrenia patients are often depressed (Buckley, Miller, Lehrer, & Castle, 2009), autism patients are often anxious (van Steensel, Bögels, & Perrin, 2011), Down syndrome patients tend to develop premature dementia (N.-C. Lee, 2017), and current depression predicts dementia later on (Manji et al., 2012; Mirza et al., 2016).

Why should faulty mitochondria be consistently involved in disorders that are so very different from one another is poorly understood. Perhaps mitochondrial dysfunction increases the vulnerability of brain cells to disease-specific factors (Devaraju & Zakharenko, 2017; Toker & Agam, 2015) such as infections, defective genes, or maternal antibodies during fetal life (P. Kramer & Bressan, 2015). Perhaps, which particular disease will turn up is determined by where the (worst) mitochondrial dysfunction happens to be located or what the nature of the dysfunction is (Devaraju & Zakharenko, 2017; Toker & Agam, 2015). What is malfunctioning, for example, might be the transport of ATP to the host cell or mitochondria’s antioxidant defense against free radicals. When forced to spend half an hour in a closed tube, mice with the first problem have an abnormally large rise of the stress hormone corticosterone and mice with the second problem an abnormally small one (Picard et al., 2015). As well, different types of mitochondrial dysfunction have different effects on neurotransmitters such as dopamine, adrenalin, noradrenalin, and serotonin (Picard et al., 2015). Notice that dysregulation of these neurotransmitters is common in mental disease; for example, excessive dopamine levels have been associated with schizophrenia (Grace, 2016).

Psychologists and psychiatrists focus on mental rather than physical health; but to uncover the cause of mental illness, cues from the body should not be overlooked. It raises a red flag when patients with a mental problem also complain of a variety of physical issues, most notably constant fatigue, or when they respond to psychotropic drugs by getting worse (Anglin, Rosebush, & Mazurek, 2012). Antipsychotics can have antiparasitic effects (Jones-Brando, Torrey, & Yolken, 2003)—which may unintentionally contribute to their success, since some forms of psychosis could be caused by parasites (P. Kramer & Bressan, 2015). Given that our bacteria-like mitochondria resemble parasites, however, it is not at all unexpected that such drugs can harm them too (Manatt & Chandra, 2011, and references therein). At recommended treatment durations and doses, drugs that directly target bacteria, such as common antibiotics, also cripple mitochondria in one or another of an assortment of ways: They wreck their outer wall, damage their DNA, mess up their quality control, and disrupt their energy production (Kalghatgi et al., 2013; Wang, Ryu, Houtkooper, & Auwerx, 2015). For similar reasons, mitochondria get damaged by exposure to any of a wide array of regularly used pesticides and other environmental pollutants (Karami-Mohajeri & Abdollahi, 2013; Meyer et al., 2013; Mostafalou & Abdollahi, 2013).

## The Art of Mitochondria Maintenance

Not all mental disorders can be cured, and at least for the time being we have no way of stopping the eventual degeneration of our body and brain. Yet we can improve our mental health and slow down our decline—if only we were willing to pay the unfashionable price at which this comes: getting enough sleep, exercising regularly, practicing relaxation techniques, eating less than we may like and less frequently, and choosing unprocessed, nutrient-rich foods.

As soon as we fall asleep, our brain cells shrink in size (Xie et al., 2013). This move—possibly a side effect of reduced firing activity—expands the space between cells by more than 60%. Throughout the brain, the extra space strikingly increases the perpetual flow of cerebrospinal fluid that flushes waste products out into the bloodstream, for eventual detoxification in the liver. In particular, the main component of Alzheimer’s plaques, beta-amyloid, is washed out of the brain twice as fast while one sleeps than while one is awake (shown in mice: Xie et al., 2013). Neurons manufacture beta-amyloid for good reasons, and in normal concentrations this substance is far from harmful (D’Andrea, 2016; Kumar, Eimer, Tanzi, & Moir, 2016; Soscia et al., 2010). First, it defends neurons against bacteria and other pathogens—some of which have been implicated in Alzheimer’s disease itself. Second, partly by helping calcium ions get into neurons when they fire, it promotes learning and memory. Still, if too much of it accumulates inside a neuron, beta-amyloid ends up harming the neuron and specifically its mitochondria (Rodrigues, Solá, Silva, & Brites, 2000)—perhaps exactly because they resemble bacteria. That beta-amyloid builds up while we are awake and is cleared while we are asleep (Huang et al., 2012) may thus be at least one of the reasons why lack of sleep damages mitochondria, interferes with learning and memory, and ultimately leads to dementia and death (Qiu et al., 2016; Xie et al., 2013; Zhao et al., 2016).

During sleep, the body and brain continue to spend energy, but the usual source of that energy, glucose, is gradually depleted and not replenished. The same happens during prolonged exercise. Mitochondria must switch to burning something else, and on that account the liver breaks down stored fat into molecules called ketone bodies. This switch means that metabolic circumstances have changed; besides acting as a circulating source of energy, ketone bodies appear to serve as carriers of this useful piece of information too (Newman & Verdin, 2014; Sleiman et al., 2016). Through the bloodstream, they reach the brain where they proceed to signal the news by regulating gene expression—targeting specifically the gene responsible for the production of BDNF. This causal chain, from exercise to ketone bodies to BDNF release, has recently been demonstrated in mice that were allowed to run on a wheel as much as they liked (Sleiman et al., 2016). Exercise actually stimulates the production of BDNF in more than one way at the same time (e.g., Sleiman et al., 2016; Wrann et al., 2013). For our ancestors, after all, physical effort tended to occur at times when one had better be smart and learn fast: when responding to danger, locating hazards, tracking prey, or exploring unfamiliar environments (Mattson, 2015; Noakes & Spedding, 2012).

Rest rusts, say the Dutch, but of course the flip side of lack of rest is a greater production of ATP and thus of free radicals. Rats that swam either 10 or 30 minutes per day had, after 20 weeks, fewer mutations in their mitochondrial DNA than rats that did nothing. However, whereas swimming daily for 10 or, much better, 30 minutes increased the number of mitochondrial DNA copies, doing it for 60 or 90 minutes markedly decreased it (Cao et al., 2012). Indeed, the benefits of regular exercise appear to outweigh its inescapable costs only so long as this is practiced at low-to-moderate intensities (Gradari, Pallé, McGreevy, Fontán-Lozano, & Trejo, 2016). Repeated mild stresses upregulate various antioxidant and repair mechanisms so that the body is better placed to cope with major stresses at a future time (Goto, Naito, Kaneko, Chung, & Radák, 2007). Note, incidentally, that this chain of events—and with it the health-promoting effects of exercise—is blocked by taking antioxidant supplements (Peternelj & Coombes, 2011).

A rule of moderation seems to apply to all forms of stress. For example, low levels of the stress hormone corticosterone, even when chronic, strengthen mitochondria but higher levels harm them (shown in rats: Markham et al., 2014). The effects of constant intense stress counteract those of BDNF, increase free-radical production, and reduce mitochondria’s capacity to create energy and lock away calcium. Practicing stress-management techniques that move one’s attention away from everyday concerns—like meditation, yoga, tai chi, or repetitive prayer—can engender positive effects on body functions within minutes. Especially in people who practice them regularly, these techniques upregulate genes that reduce free-radical damage, downregulate those that foster inflammation, and support the production of ATP and its use by cells (Bhasin et al., 2013).

Similar benefits as those brought about by getting enough sleep and exercising, and at least partly via the same glucose-depletion mechanism, can be achieved by eating enough but less than one would be spontaneously inclined to do (Gano, Patel, & Rho, 2014; Maalouf, Rho, & Mattson, 2009; Mattson, 2012). Evolutionarily speaking, protracted food restriction may signal famine and dictate that energy be directed away from current reproduction efforts and toward maintenance and repair mechanisms that will allow one to reproduce at a future, more appropriate time. It is unclear whether the restriction that matters is that of calories or meal frequency (Longo & Panda, 2016), of specific food categories, such as carbohydrate-dense products like flour and sugar (Spreadbury, 2012), or of specific food components, such as the amino acid methionine in proteins (McIsaac, Lewis, Gibney, & Buffenstein, 2016). Most of these regimens raise the level of ketone bodies, which have beneficial effects quite apart from their ability to recruit BDNF. Besides promoting mitochondrial reproduction and energy generation, ketone bodies protect cells, including neurons, from an impressive variety of insults (Newman & Verdin, 2014). The so-called ketogenic diet contains a good deal of fat and some protein but very little carbohydrate; the unavailability of glucose (which chiefly comes from carbohydrate but can also be created from protein) massively raises the levels of ketone bodies in the blood. The diet has a long history of success in treating epilepsy in children—even when they do not respond to drugs—with benefits that in most cases appear to linger after its discontinuation (Martinez, Pyzik, & Kossoff, 2007). A multitude of other neurological and nonneurological conditions have been reported to get better with a ketogenic diet, including autism (Castro et al., 2015), Alzheimer’s and Parkinson’s disease, traumatic brain injury, and stroke (Barañano & Hartman, 2008; Gano et al., 2014).

Food can be scarce, and we may not have evolved a great desire to restrict our diets. Whether or not we manage to eat less, however, and more important, we can feed our mitochondria those vitamins, minerals, enzymes, cofactors, polyphenols, and other nutrients they need to do their job (Liu & Ames, 2005; Parikh et al., 2009). In combinations and concentrations that, unlike in supplements (Villanueva & Kross, 2012), tend to be harmless, some or other of these crucial substances can be found in unprocessed natural foods. These foods include fruit and vegetables, fish, shellfish and seaweeds, meats and organ meats like liver and heart, nuts and seeds, and fermented fare. Vitamin D, a hormone that forms rapidly in the unprotected skin during sunbathing, should be high on the list too. In people with low levels of vitamin D in the blood, vitamin D supplements make mitochondria work better, possibly by regulating the entry of calcium in them (Sinha, Hollingsworth, Ball, & Cheetham, 2013). Adoption of a lifestyle designed to help mitochondrial function, pivoting on an outstandingly nutrient-rich diet, has been reported to have gotten a physician with progressive multiple sclerosis out of her wheelchair (Wahls, 2011).

Properly feeding one’s mitochondria could even improve one’s social rank—as suggested by a study on rats. In rats, just like in humans, motivation and social competition are regulated by the nucleus accumbens, a brain structure whose malfunctioning contributes to depression and anxiety (Bewernick et al., 2010; Chen, Rada, Bützler, Leibowitz, & Hoebel, 2012; Levita, Hoskin, & Champi, 2012). Anxious rats have lower concentrations of ATP (implying worse-functioning mitochondria) in their nucleus accumbens than less anxious ones, and they tend to become subordinate to them during social encounters (Hollis et al., 2015). The causal nature of the connection between malfunctioning mitochondria and submissiveness was exposed by treating some rats’ nucleus accumbens with drugs that happen to either inhibit or enhance mitochondrial energy production. When two equally anxious male rats met, the one whose mitochondria had been inhibited was more likely than the other to become submissive—although not more likely to become less sociable or sick in any way. Conversely, an anxious rat that would normally have surrendered to a less anxious one became as likely as the other to play first fiddle when its nucleus accumbens was infused with vitamin B3, a stimulator of mitochondrial function (Hollis et al., 2015). Individual differences in social behavior are thus regulated, in rats, by how well the mitochondria work in a brain structure that is also present with a similar function in humans. It does not seem too farfetched to speculate that the unwillingly submissive among us might be able to become more assertive, and perhaps less depressed, if they supply their own mitochondria with the nutrients they need.

We tend to think of ourselves as human beings and human beings only: Yet 38 trillion microbes (Sender, Fuchs, & Milo, 2016), distributed into at least 2,172 known species (Hugon et al., 2015), populate each of us in places as supremely personal as our mouth, armpits, gut, genitals—and brain (Branton et al., 2013). It is a sobering thought that we house at least as many foreign as human cells (Sender et al., 2016), and those cells that we consider human stem from archaea and bacteria. With the single exception of red blood cells, which got rid of their nucleus too, each of these “human” cells is itself densely inhabited by direct descendants of bacteria in the form of mitochondria. They have made themselves indispensable and this is good and bad news. On the one hand, mitochondria *make* our mental health—besides furnishing the energy for the brain to function at all, they enable synaptic plasticity, produce hormones and signaling molecules, dish neurotransmitters out and rein neurotransmitters in. On the other, they also *break* it—even their proper operation corrodes our brain, while their malfunctioning is associated with cognitive deficits, intellectual disabilities, neurodegenerative disorders, and mental illness. Whether as victims or as perpetrators, mitochondria are right in the middle of virtually all human afflictions. They still look a little like their bacterial forefathers and still retain a bit of independence from us. But because of the deal they struck with archaea 2 billion years ago, their health is now entwined with ours. So, to help get the best out of us as humans, we may actually want to do what is best for our bacteria-like components: exercise, sleep, spend time in the sun, eat well, and meditate.
